# Zinc, Copper, and Manganese Supplementations in Rheumatic Disease: A Narrative Review

**DOI:** 10.31138/mjr.270325.amr

**Published:** 2025-08-26

**Authors:** Jozélio Freire de Carvalho, Ana Tereza Amoedo Martinez

**Affiliations:** 1 Núcleo de Pesquisa em Doenças Crônicas não Transmissíveis (NUPEC), School of Nutrition from the Federal University of Bahia, Salvador, Bahia, Brazil;; 2 Universidade Estadual de Feira de Santana, Feira de Santana, Bahia, Brazil;; 3 Novaclin, Grupo CITA, Salvador, Bahia, Brazil

**Keywords:** supplementations, diseases, rheumatoid arthritis, rheumatic

## Abstract

**Background:**

Trace elements, including zinc (Zn), copper (Cu), and manganese (Mn), play crucial roles in various biological functions, particularly in oxidative stress regulation and immune response. Their potential involvement in rheumatic diseases has been suggested, with evidence indicating altered levels of these elements in conditions such as rheumatoid arthritis (RA) and systemic lupus erythematosus (SLE). However, the efficacy of supplementation remains unclear.

**Objective:**

This narrative review aims to evaluate the available evidence on Zn, Cu, and Mn supplementation in patients with rheumatic diseases.

**Methods:**

A comprehensive literature search was conducted in PubMed, Scielo, and LILACS databases for studies published from 1965 to April 2024. The search followed PRISMA guidelines and included randomised controlled trials (RCTs) and observational studies investigating Zn, Cu, or Mn supplementation in rheumatic diseases.

**Results:**

A total of four studies met the inclusion criteria, focusing on Zn and Cu supplementation. No studies on Mn supplementation were identified. Among the included studies, two evaluated Zn supplementation in RA and Behçet’s disease, while two assessed Cu supplementation in RA and SLE. The mean participant age ranged from 35 to 54.3 years, with female predominance (28% to 89%). Follow-up durations varied from 4 to 24 weeks. Zn supplementation demonstrated clinical and immunological benefits, including reduced disease activity in Behçet’s disease and improved inflammatory markers in RA. Conversely, Cu supplementation did not show significant therapeutic effects. Reported side effects were mild and primarily gastrointestinal, occurring in 4% to 33% of participants.

**Conclusion:**

Zn supplementation appears beneficial in certain rheumatic diseases, particularly in RA and Behçet’s disease, while Cu supplementation lacks significant therapeutic effects. Further high-quality RCTs are needed to establish definitive recommendations.

## INTRODUCTION

Trace elements, including Zn, Cu, and Mn, are essential micronutrients involved in various physiological processes, including immune function, oxidative stress regulation, and enzymatic activities. Zn and Cu, in particular, are critical components of superoxide dismutase (SOD), a key antioxidant enzyme implicated in inflammatory and autoimmune diseases.^1^

Several studies have suggested altered trace element levels in patients with rheumatic diseases. Meta-analyses indicate that RA patients exhibit increased serum Cu and decreased serum Zn levels compared to healthy controls.^2^ Similarly, hair analysis studies in RA patients reveal lower Zn, Cu, and Mn content in both smokers and non-smokers.^3^ Despite these observations, clinical studies evaluating the impact of Zn, Cu, or Mn supplementation on rheumatic diseases remain limited.

This narrative review aims to summarise the current evidence regarding Zn, Cu, and Mn supplementation in rheumatic diseases, focusing on their potential therapeutic roles and clinical outcomes.

## METHODS

A literature search was conducted in PubMed, Scielo, and LILACS databases for studies published between 1965 and April 2024. The search strategy included terms related to “zinc supplementation,” OR “copper supplementation,” OR “manganese supplementation,” AND “rheumatic diseases” OR “rheumatoid arthritis” OR “systemic lupus erythematosus” OR “Sjogren’s syndrome” OR “myositis” OR “spondyloarthritis” OR “Behçet’s disease” OR “vasculitis”. The inclusion criteria were as follows:

- Studies evaluating Zn, Cu, or Mn supplementation in rheumatic diseases
- Randomised controlled trials (RCTs) or observational studies
- Outcomes related to disease activity, inflammatory markers, or clinical symptoms

The exclusion criteria were “in vivo” and “in vitro” studies, case report, case series, review articles, editorial.

## RESULTS

A total of four studies met the inclusion criteria (**Table 1**). No studies on Mn supplementation were identified. Two studies investigated Zn supplementation, while two focused on Cu. The mean participant age ranged from 35 to 54.3 years, with a predominance of female participants (28%–89%).

**Table 1.** Studies on zinc and copper supplementation in rheumatic diseases.

**Author, reference**	**Study design**	**Country**	**N, age, gender**	**Rheumatic disease**	**Disease duration**	**Treatment regimen**	**Follow-up**	**Outcome**	**Side effects**
Faghfouri et al., 2022.^4^	Double-blind placebo-controlled randomized clinical trial	Iran	50 43 ± 11.18 yo 28% females	Behçet’s disease	ND	Zinc gluconate (30 mg/day) or placebo	12 weeks	Zn reduced nonocular Iranian Behçet's disease dynamic activity measure score. Zn decreased mRNA and protein expression of TLR-2.	4% (mild gastrointestinal)
Simkin et al., 1976^5^	Double-blind placebo-controlled	United States	24 54.3 ± 11.2 yo ND	Rheumatoid arthritis	11.3 ± 6.7 years	Zinc sulfate (220mg thrice a day) or placebo	12 weeks	Zn group improved joint swelling and tenderness, morning stiffness, walking time, and patient overall evaluation.	3/12 (25%) had vomit, and 5/12 (33%) had constipation. The others were higher in placebo.
Duffy et al., 2004^6^	Double-blind placebo-controlled factorial trial	United Kingdom	52 46.6 (22–76) yo 89% females	Systemic lupus erythematosus	ND	Copper diglycinate 3mg/day vs. omega-3 vs. combination	24 weeks	No effect of Cu. The omega-3 group reduced disease activity.	ND
DiSilvestro et al., 2020^7^	Open, prospective placebo-controlled trial	United States	23 and 48 controls 35–53 yo 74% females	Rheumatoid arthritis	ND	Cu glycine 2mg	4 weeks	Cu did not affect ceruloplasmin, which is an acute phase reagent.	ND

## ZINC SUPPLEMENTATION

Two studies evaluated Zn supplementation:

- Behçet’s disease: A double-blind, placebo-controlled RCT by Faghfouri et al. (2022) [4] demonstrated that Zn gluconate (30 mg/day) significantly reduced disease activity scores and decreased Toll-like receptor (TLR) expression.
- Rheumatoid arthritis: A study by Simkin et al. (1976)^5^ found that Zn sulfate (220 mg thrice daily) led to improvements in joint swelling, morning stiffness, and patient-reported outcomes.

## COPPER SUPPLEMENTATION

Two studies assessed Cu supplementation:

Systemic lupus erythematosus: A trial by Duffy et al. (2004)^6^ found no significant effect of Cu di-glycinate (3 mg/day) on disease activity, whereas omega-3 supplementation was beneficial.

Rheumatoid arthritis: DiSilvestro et al. (2020)^7^ reported that Cu supplementation (2 mg/day) did not alter ceruloplasmin levels, suggesting limited impact on inflammation.

## SAFETY AND SIDE EFFECTS

Two studies reported adverse effects. Zn supplementation led to mild gastrointestinal symptoms in 4% of participants in one study, while another reported vomiting (25%) and constipation (33%). No severe adverse effects were documented.

## DISCUSSION

This narrative review highlights the potential role of Zn and Cu supplementation in rheumatic diseases, particularly in rheumatoid arthritis and Behçet’s disease. Zn supplementation demonstrated clinical benefits, including reduced disease activity and improvement in inflammatory markers.^4,5^ However, Cu supplementation did not exhibit significant effects in RA or lupus, suggesting that its role in these conditions might be limited.^6,7^ The findings align with previous studies indicating that Zn plays a crucial role in immune modulation and inflammatory response, whereas Cu’s involvement in rheumatic disease pathophysiology remains unclear.^1,2^

Zinc and copper play pivotal roles in inflammatory and autoimmune processes through distinct molecular mechanisms that influence immune responses and oxidative stress. Zinc modulates inflammation by inhibiting the NF-κB signalling pathway, leading to reduced expression of pro-inflammatory cytokines such as TNF-α and IL-1β. It also upregulates anti-inflammatory mediators like A20 and PPAR-α, contributing to the attenuation of inflammatory responses. Furthermore, zinc functions as an antioxidant by competing with redox-active metals like iron and copper for binding sites on cell membranes, thereby preventing the formation of highly reactive free radicals.^8^ Copper, essential for the activity of antioxidant enzymes such as superoxide dismutase (SOD), also influences immune modulation by affecting T-cell proliferation and cytokine production. However, excessive copper exposure can dysregulate inflammatory pathways by activating signalling cascades like NF-κB and MAPKs, promoting the release of inflammatory cytokines and contributing to the pathogenesis of autoimmune diseases.^8,9^ Maintaining a proper balance of these trace elements is crucial for preserving immune homeostasis and preventing chronic inflammatory and autoimmune conditions.

The Faghfouri study^4^ was a double-blind, randomised, placebo-controlled clinical trial that is methodologically robust, the sample size is not widely detailed, limiting generalisability. The research suggests immunomodulatory benefits of zinc, but larger sample studies are needed to confirm its clinical effects. Regarding Simkin trial,^5^ it presents some methodological limitations, such as a small sample size and insufficient control over external variables. The study calls for replications with larger and more rigorous samples. In regard to Duffy study,^6^ focusing on omega-3 and copper supplementation in patients with lupus, the study presents a controlled design with multiple groups, allowing detailed comparisons. However, the small sample size and lack of longitudinal data limit the long-term interpretation of the effects. At last, the DiSilvestro article^7^ examines the effect of copper on biomarkers such as ceruloplasmin in rheumatoid arthritis, offering a precise biochemical analysis. However, the absence of a placebo control and the small sample size limit conclusions on clinical efficacy.

In summary, the studies vary considerably in terms of methodological rigor. While the more recent trials^4^ employ more robust methodologies, older studies, like Simkin,^5^ suffer from limitations in experimental control. The use of biomarkers, as seen in Duffy et al.^6^ and DiSilvestro et al.,^7^ adds depth to the investigations, but the lack of control over dietary and lifestyle factors in some studies hampers interpretation. The main implication of these studies is the need for more rigorous research with larger samples and better control.

One of the main limitations of this review is the small number of available studies, which restricts the generalisability of the findings. The heterogeneity in study design, sample sizes, and supplementation dosages further complicates direct comparisons between studies.^3^ Additionally, the follow-up periods varied widely, making it difficult to determine long-term effects and potential late-onset adverse events. The lack of manganese studies is another major limitation, as this trace element may also play a role in inflammatory and autoimmune conditions.^1^

Another concern is the variability in the baseline characteristics of participants, including disease duration, severity, and prior treatments, which could have influenced the outcomes. Some studies lacked sufficient detail on these aspects, making it difficult to assess whether supplementation was more effective in early or advanced disease stages.^4^ Furthermore, the differences in formulations and bioavailability of Zn and Cu supplements may have impacted their efficacy, as not all forms of these trace elements are equally absorbed and utilised by the body.^5,6^

Despite these limitations, this review has several strengths. It provides a comprehensive synthesis of the current evidence on Zn and Cu supplementation in rheumatic diseases. Furthermore, the inclusion of randomised controlled trials enhances the reliability of the findings. The analysis of side effects also offers valuable insight into the safety profile of these supplements, which is crucial for clinical recommendations. The reported adverse events were mild and mostly gastrointestinal, suggesting that Zn and Cu supplementation is generally safe when administered in appropriate doses.^4,7^

Future research should focus on larger, well-controlled trials with longer follow-up periods to assess the sustained benefits and risks of Zn and Cu supplementation. It would be particularly useful to investigate the potential synergistic effects of these trace elements with other dietary interventions or pharmacological treatments. Moreover, studies should explore the molecular mechanisms underlying their effects, particularly how Zn modulates inflammatory pathways and immune cell function in rheumatic diseases.^1,2^

Lastly, personalised medicine approaches could enhance the effectiveness of trace element supplementation by identifying subgroups of patients who may benefit the most based on genetic, metabolic, or immune profiles. Understanding individual variations in trace element metabolism could lead to more targeted therapeutic strategies, ultimately improving patient outcomes in rheumatic diseases.^6^ Overall, while the current evidence suggests a potential benefit of Zn supplementation, more robust and well-designed studies are needed to establish definitive clinical guidelines.

## CONFLICT OF INTEREST

The author has no conflicts of interest to declare.

## FUNDING

None.
